# Genome-based classification of the *Streptomyces violaceusniger* clade and description of *Streptomyces sabulosicollis* sp. nov. from an Indonesian sand dune

**DOI:** 10.1007/s10482-021-01564-0

**Published:** 2021-04-02

**Authors:** Ali B. Kusuma, Imen Nouioui, Michael Goodfellow

**Affiliations:** 1 School of Natural and Environmental Sciences, Newcastle University, Ridley Building 2, Newcastle upon Tyne, NE1 7RU UK; 2 Indonesian Centre for Extremophile Bioresources and Biotechnology (ICEBB), Faculty of Biotechnology, Sumbawa University of Technology, Sumbawa Besar, 84371 Indonesia; 3 Leibniz-Institut DSMZ – German Collection of Microorganisms and Cell Cultures, Inhoffenstraße 7B, 38124 Braunschweig, Germany

**Keywords:** *Streptomyces sabulosicollis*, Polyphasic taxonomy, *Streptomyces violaceusniger* clade, Genomics, Genome mining

## Abstract

A polyphasic study was designed to determine the taxonomic provenance of a strain, isolate PRKS01-29^T^, recovered from an Indonesian sand dune and provisionally assigned to the *Streptomyces violaceusniger* clade. Genomic, genotypic and phenotypic data confirmed this classification. The isolate formed an extensively branched substrate mycelium which carried aerial hyphae that differentiated into spiral chains of rugose ornamented spores, contained LL-as the wall diaminopimelic acid, MK-9 (H_6_, H_8_) as predominant isoprenologues, phosphatidylethanolamine as the diagnostic phospholipid and major proportions of saturated, *iso*- and *anteiso*- fatty acids. Whole-genome sequences generated for the isolate and *Streptomyces albiflaviniger* DSM 41598^T^ and *Streptomyces javensis* DSM 41764^T^ were compared with phylogenetically closely related strains, the isolate formed a branch within the *S. violaceusniger* clade in the resultant phylogenomic tree. Whole-genome sequences data showed that isolate PRKS01-29^T^ was most closely related to the *S. albiflaviniger* strain but was distinguished from the latter and from other members of the clade using combinations of phenotypic properties and average nucleotide identity and digital DNA:DNA hybridization scores. Consequently, it is proposed that isolate PRKS01-29^T^ (= CCMM B1303^T^ = ICEBB-02^T^ = NCIMB 15210^T^) should be classified in the genus *Streptomyces* as *Streptomyces sabulosicollis* sp. nov. It is also clear that streptomycetes which produce spiral chains of rugose ornamented spores form a well-defined monophyletic clade in the *Streptomyces* phylogenomic tree., the taxonomic status of which requires further study. The genome of the type strain of *S. sabulosicollis* contains biosynthetic gene clusters predicted to produce new natural products.

**Supplementary Information:**

The online version contains supplementary material available at 10.1007/s10482-021-01564-0.

## Introduction

The classification of *Streptomyces* species is especially challenging given the high number of validly published species (https://www.bacterio.net.streptomyces.html), the limited resolution of 16S rRNA gene sequences in their delineation (Labeda et al. 2012, 2017) and evidence that the genus is underspeciated (Yamac et al. 2011; Hamm et al. 2017). However, multi-locus sequence analyses (MLSA) of concatenated protein-coding house-keeping genes (Ayed et al. 2018; Kusuma et al. 2020; Li et al. 2020; Martinet et al. 2020) and comparative surveys of whole-genome sequences (Nouioui et al. 2018) provide invaluable data for the circumscription of novel *Streptomyces* species. MLSA analyses have revealed a correlation between the delineation of phylogenetic clades and associated phenotypic properties (Rong and Huang 2014; Labeda et al. 2014), as exemplified by the assignment of streptomycetes with spiral chains of rugose ornamented spores to a well supported taxon (Labeda et al. 2017), known as the *Streptomyces violaceusniger* clade (Sembiring et al. 2000; Kumar and Goodfellow 2008, 2010). Representatives of this clade show the same pattern of HPLC-detected metabolites (Ward and Goodfellow 2004; Goodfellow et al. 2007), give a characteristic amplification product with taxon-specific primers (Kumar et al. 2007) and form a characteristic grey aerial spore mass and a greyish yellow substrate mycelium on oatmeal agar (International *Streptomyces* Project medium 3 [ISP 3]., Shirling and Gottlieb 1966) (Sembiring et al. 2000; Kumar and Goodfellow 2008, 2010; Goodfellow et al. 2007)

Improvements in the classification of the *S. violaceusniger* clade (Rong and Huang 2012; Komaki et al. 2017; Labeda et al. 2017; Zhou et al. 2017) led to the recognition of 16 species which include *Streptomyces albiflaviniger* (Goodfellow et al. 2007, Euzéby 2008), *Streptomyces himastatinicus* (Kumar and Goodfellow 2008), *Streptomyces hygroscopicus* (Jensen 1931) Waksman and Henrici 1948*, Streptomyces iranensis* (Hamedi et al. 2010), *Streptomyces javensis* (Sembiring et al. 2000, 2001), *Streptomyces malaysiensis* (Al-Tai et al. 1999), *Streptomyces melanosporofaciens* (Arcamone et al. 1959), *Streptomyces rapamycinicus* (Kumar and Goodfellow 2008), *Streptomyces rhizosphaericus* (Sembiring et al. 2000, 2001), *Streptomyces solisilvae* (Zhou et al. 2017) and *Streptomyces violaceusniger* corrig (Waksman and Curtis 1916) Pridham et al. 1958, as emended by Labeda and Lyons (1991), the earliest validly published species in the taxon. An additional species, “*Streptomyces ruani*” (Kumar and Goodfellow 2008) was shown to be invalid by Tindall (2014). Strains assigned to the clade have been detected in diverse habitats (Kumar et al. 2007) but are usually associated with rhizosphere and non-rhizosphere soil (Sembiring et al. 2000; Sahin et al. 2010).

Strains classified in the *S. violaceusniger* clade have an impressive track record as a source of new antibiotics (DeBoer et al. 1970; Chen et al. 2003; Cheng et al. 2010; Xie et al. 2019), antiparasitic metabolites (Sun et al. 2002), antitumour compounds (Lam et al. 1990; Wang et al. 2013), enzymes (Rabe et al. 2017) and immunosuppressants (Vezina et al. 1975) and biocontrol agents (Clermont et al. 2010; Palaniyandi et al. 2016; Sarwar et al. 2019) hence the continued interest in them for genome mining and natural product discovery. Members of this taxon are gifted in the sense of Baltz (2017) as they have large genomes (> 8 Mbp) rich in biosynthetic gene clusters (BGCs) predicted to encode for specialised metabolites (Baranasic et al. 2013; Horn et al. 2014; Komaki et al. 2018). Prospecting for *Streptomyces* diversity also shows that sampling strains from unexplored, including extreme habitats, raises the probability of finding new compounds (Nicault et al. 2020) and that streptomycete genomes are a prolific source of novel BGCs (Vicente et al. 2018; Martinet et al. 2020).

The present study was designed to classify a putative new member of the *S. violaceusniger* clade based on genomic, genotypic and phenotypic data and to gain an insight into its potential as a source of new specialised metabolites. The resultant datasets showed that the isolate represents a novel species, named *Streptomyces sabulisicollis* sp. nov. Associated phylogenomic data clarified the internal taxonomic structure of the *S. violaceusniger* clade and relationships to its closest phylogenetic neighbours.

## Materials and methods

### Isolation, maintenance and cultivation

Isolate PRKS01-29^T^ was isolated from an arid, non-saline soil sample (pH 5.8., organic matter content 0.06%) collected just below the surface of a sand dune in the Parangkusumo Region (8° 1′7 513″ S/ 110° 19′ 11.04″ E) of Yogyakarta Province, Java, Indonesia following incubation on Actinomycete Isolation Agar (HiMedia, Einhausen, Germany), pH 7.3, supplemented with cycloheximide (50 µg/mL), nalidixic acid (25 µg/mL) and nystatin (25 µg/mL) and incubated for 7 days at 45 °C, as described previously (Kusuma et al. 2020). The isolate and *S. albiflaviniger* DSM 41598^T^*, S. iranensis* DSM 41954^T^*, S. javensis* DSM 41764^T^*, S. malaysiensis* NBRC 13472^T^*, S. rapamycinicus* NRRL 5491^T^ and *S. rhizosphaericus* NRRL B-24304^T^ and *S. violaceusniger* DSM 40583^T^ were maintained on yeast extract-malt extract agar (International *Streptomyces* Project medium 2 [ISP 2]., Shirling and Gottlieb 1966) and as mixtures of hyphal fragments and spores in 20%, v/v glycerol at −20 °C and −80 °C. The type strains of *S. albiflaviniger* and *S. iranensis* were obtained from the Leibniz Institute DSMZ German Collection of Microorganisms and Cell Cultures GmbH, Braunschweig, Germany and the remaining reference strains were from the personal collection of Professor Michael Goodfellow, Newcastle University, Newcastle-upon-Tyne, United Kingdom. Biomass for the chemotaxonomic studies carried out on the isolate was prepared in 1L Erlenmeyer flasks containing 250 mL of sterile ISP 2 broth (Shirling and Gottlieb 1966), the flasks were shaken at 180 rpm for 14 days at 28 °C and the resultant biomass harvested by centrifugation at 4000 rpm for 10 min, washed twice in sterile distilled water and freeze dried for 3 days.

### Acquisition of chemotaxonomic, cultural and morphological properties

The isolate was examined for chemotaxonomic, cultural and morphological properties of value in *Streptomyces* systematics (Kämpfer 2012; van der Aart et al. 2019). Gram-stain (Hucher’s modification, Society for American Bacteriology 1957) and micromorphological features were recorded following growth on ISP 3 agar for 7 days at 28 °C. Growth from the ISP 2 preparation was examined for spore-chain arrangement and spore-surface ornamentation using a scanning electron microscope (Tescan Vega 3, LMU instrument) and the procedure described by O’Donnell et al. (1993). The ability of the test and associated marker strains to grow at different temperatures, pH regimes and in the presence of various concentrations of sodium chloride was carried out in triplicate, as mentioned by Kusuma et al. (2020). Standard chromatographic methods were used to detect the isomers of diaminopimelic acid (A_2_pm) (Staneck and Roberts 1974), whole-organism sugars (Lechevalier and Lechevalier 1970) and for menaquinones and polar lipids by applying the integrated procedure of Minnikin et al. (1984), using appropriate controls. Cellular fatty acids were extracted from freeze dried cells of the isolate and fatty acid methyl esters (FAMES) prepared following saponification and methylation using the procedure described by Miller (1982), as modified by Kuykendall et al. (1988). The FAMES were separated by gas chromatography (Agilent 68,908 instrument), the resulted peaks automatically integrated and the fatty acid names and properties determined using the standard Microbial Identification (MIDI) system, version 4.5 and the ACTIN 6 database (Sasser 1990). The growth and cultural characteristics of the isolate and reference strains were determined on tryptone yeast extract, yeast extract-malt extract, oatmeal, inorganic salts-starch, glycerol-asparagine, peptone-yeast extract-iron and tyrosine agar plates (ISP media 1–7; Shirling and Gottlieb 1966) for 21 days at 28 °C., aerial spore mass and substrate mycelial colours and those of diffusible pigments were recorded using colour charts (Kelly 1958).

### Whole genome sequencing

Genomic DNA was extracted from wet biomass of single colonies of the isolate, *S. albiflaviniger* DSM 41598^T^ and *S. javensis* DSM 41764^T^, grown on ISP 2 agar for 7 days at 28 °C, following the protocol provided by MicrobesNG (Birmingham, UK) (http://www.microbesng.uk) and sequenced on an Miseq instrument (Illumina, San Diego, USA). The quality of the extracted DNA preparations and the sequencing of genomic DNA libraries was achieved, as described by Kusuma et al. (2020). The libraries were sequenced following the 2 × 250-bp paired-end protocol (MicrobesNG, Birmingham, UK). Reads under 200 bp were discarded and contigs assembled using SPAdes software version 3.1.1 (Bankevich et al. 2012). The draft genome assemblies of the strains were annotated using the RAST-SEED web server (Aziz et al. 2008; Overbeek et al. 2014) with default options and are available from GenBank database.

### Phylogeny

An almost complete 16S rRNA gene sequence (1454 nucleotides [nt]) (GenBank accession number MK503616) was taken directly from the draft genome of the isolate using the ContEst16S tool from the EZBioCloud webserver (https://www.ezbiocloud.net/tools/contest16s) (Lee et al. 2017); this had been compared with the associated 16S rRNA gene sequence generated using Sanger method. The gene sequence was aligned with corresponding sequences of the most closely related type strains of *Streptomyces* species retrieved from the EzBiocloud webserver (Yoon et al. 2017) using MUSCLE software (Edgar 2004). Pairwise sequence similarities were determined using the single-gene tree option from the Genome-to-Genome Distance Calculator (GGDC) webserver (Meier-Kolthoff et al. 2013a, b). Phylogenetic trees were inferred using the maximum-likelihood (ML., Felsenstein 1981), maximum-parsimony (MP., Fitch 1971) and neighbour-joining (NJ., Saitou and Nei 1987) algorithms. A ML tree was inferred from alignments with RAxML (Stamatakis 2014) using rapid bootstrapping with the auto Maximum-Relative-Error (MRE) criterion (Pattengale et al. 2010) and a MP tree was constructed from the alignments with the Tree Analysis New Technology (TNT) program (Goloboff et al. 2008) using 1000 bootstraps together with tree-bisection-and-reconnection branch swapping and ten random sequence replicates. The sequences were checked for computational bias using the X2 test from PAUP*(Phylogenetic Analysis Using Parsimony) (Swofford 2002).The trees were evaluated using bootstrap analyses based on 1000 replicates (Felsenstein 1985) from the MEGA X software package (Kumar et al. 2018) and the two-parameter model of Jukes and Cantor (1969) then rooted with the 16S rRNA gene sequence from *Streptomyces albus* subsp. *albus* NRRL B-1811^ T^ (GenBank accession number JX486031.1), the type strain of the type species of the genus *Streptomyces*.

### Comparison of genomes

The draft genome sequences generated for isolate PRKS01-29^T^, *S. albiflaviniger* DSM 41598^T^ and *S. javensis* DSM 41764^T^ were compared with corresponding sequences of type strains of species classified in the *S. violaceusniger* 16S rRNA gene clade. The ML phylogenomic tree inferred using the codon tree option in the PATRIC webserver (Wattam et al. 2017), which was based on aligned amino acids and nucleotides derived from 453 single copy genes in the genome dataset matched against the PATRIC PGFams database (http://www.patricbrc.org), was generated using the RAxML algorithm (Stamatakis 2006). The genome sequences of isolate PRKS01-29^T^ and the *S. albiflaviniger* and *S. javensis* strains were compared with one another and with those of *S. antimycoticus* NRRL B-24289^T^, *S. himastatinicus* ATCC 53653^T^, *S. hygroscopicus* subsp. *hygroscopicus* NBRC 16556^T^, *S. iranensis* DSM 41954^T^*, S. malaysiensis* DSM 4137^T^*, S. melanosporofaciens* DSM 40318^T^*, S. milbemycinicus* NRRL 5739^T^*, S. rapamycinicus* NRRL 5491^T^, *S. rhizosphaericus* NRRL-24304^T^, *S. sparsogenes* DSM 40356^T^ and *S. violaceusniger* DSM 40503^T^. Average nucleotide identity (orthoANI., Lee et al. 2016) and digital DNA-DNA hybridisation (dDDH., Meier-Kolthoff et al. 2013a) values were determined between the isolate and members of the *S. violaceusniger* clade using the ANI calculator from the EzBioCloud (https://www.ezbiocloud.net/tools/ani) and the GGDC webserver (http://ggdc.dsmz.de/ggdc), respectively. The presence of natural product-BGCs in the genome of the strains were detected using the antiSMASH 5.0 platform (Blin et al. 2019) with default option available at https://antismash.secondarymetabolites.org.

### Phenotypic tests

Isolate PRKS01-29^T^ and the type strains of its closest phylogenomic neighbours were examined for phenotypic properties that distinguish between species classified in the *S. violaceusniger* 16S rRNA gene clade (Sembiring et al. 2000; Goodfellow et al. 2007; Kumar and Goodfellow 2008, 2010; Hamedi et al. 2010; Zhou et al. 2017). Biochemical, degradation and physiological properties were acquired using media and methods described by Williams et al. (1983) and enzyme profiles with API-ZYM strips (BioMériux, France). All of the tests were carried out in duplicate using a standard inoculum equivalent to 5.0 on the McFarland scale (Murray et al. 1999).

### Screening for bioactivity

The isolate was screened for antimicrobial activity against a panel of wild type microorganisms (primary screens) and *Bacillus subtilis* reporter strains (secondary screens) using a standard plug assay (Fiedler 2004). Plugs of isolate PRKS01-29^T^ were taken from ISP 2 and ISP 3 agar (Shirling and Gottlieb 1966) and from MMM and from 410 agar (Goodfellow and Fiedler^2010^) plates incubated for 14 days at 28 °C and added to cultures of wild type strains of *Bacillus subtilis, Candida albicans*,* Escherichia coli*,* Micrococcus luteus*,* Pseudomonas aeruginosa* and *Staphylococcus aureus*., all of the strains were obtained from Public Health Laboratory Service, Freeman Hospital, Newcastle-upon-Tyne, United Kingdom*.* The wild type strains were prepared by inoculating 500 μL of overnight cultures grown at 37 °C in 25 mL Luria Bertani (LB) broth (Sigma Aldrich, UK) to an optical density (OD) of 0.6 and the resultant preparations diluted to give an OD value of 0.0125 by mixing 100 mL of the LB media with the same proportion of nutrient agar (Sigma Aldrich, UK)., each of the resultant preparations was carefully mixed, poured into the square Petri dishes containing the agar plugs of the isolate and the plate incubated overnight at 37 °C. The incubated plates were observed for the presence and sizes (in millimetres) of inhibition zones around the agar plugs. In the secondary assays, agar plugs were added to overnight cultures of six *B. subtilis* reporter strains grown as described above., the reporter strains were designed to detect modes of action of antimicrobial compound(s) produced by the isolate, as shown in Table S1. Overnight cultures of the strains were grown at 37 °C in Luria Bertani broth then mixed with a similar volume of nutrient agar (Sigma-Aldrich, UK) to give an optical density reading of 0.0125. The resultant preparations were examined for the presence of blue halos around the circumference of inhibition zones, the latter are formed when bioactive compound(s) produced by the isolate cleave X-gal in the agar media to 5-bromo-4-chloro-3-hydroxy indole (blue compound) and galactose.

## Results and discussion

The chemotaxonomic, colonial and morphological properties of the isolate showed that it was a *bona fide* member of the *S. violaceusniger* clade (Sembiring et al. 2000; Goodfellow et al. 2007; Kumar and Goodfellow 2008, 2010; Hamedi et al. 2010; Nguyen and Kim 2015; Zhou et al. 2017). The organism was found to be aerobic, Gram-stain positive, formed an extensively branched substrate mycelium and aerial hyphae that differentiated into spiral chains of rugose ornamented spores (Fig S1), produced a dark grey to black aerial spore mass and a grey yellow substrate mycelium on oatmeal agar (Fig S2), contained LL-A2pm as the diamino acid of the peptidoglycan, MK-9 (H_6_) (58.4%) and MK-9 (H_8_) (41.6%) as the predominant isoprenologues, galactose, glucose, mannose and ribose as whole cell sugars and gave a polar lipid profile consisting of diphosphatidylglycerol, two phosphatidylglycerols, phosphatidylinositol, two phosphatidylinositol mannosides and two unknown phospholipids (Fig S3).

The major fatty acids (> 10%) of the isolate were *iso*-C_15:0_ (14.4%), *anteiso*-C_15:0_ (13.8%) and *iso*-C_16:0_ (27.2%) with lower proportions of *iso*-C_14:0_ (4.9%), C_14:0_ (1.0%), *iso*-H-C_16:1_ (1.2%), C_16:0_ (9.3%), *anteiso*-ω9c-C_17:1_ (1.8%), *iso*-C_17:0_ (6.5%), *anteiso*-C_17:0_ (9.9%), cyclo C_17:0_ (1.9%), C_17:0_ (1.1%), C_16:1-_ω7c/ C_16:1-_ω6c (1.2%) and iso-C_17:1_ ω9c/10-methyl C_16:0_ (2.8%)., trace components made up the balance of the profile. Complex mixtures of saturated straight chain and *iso*- and *anteiso*- fatty acids have been reported for the type strains of *S. fabae* (Nguyen and Kim 2015), *S. iranensis* (Hamedi et al. 2010), *S. malaysiensis* (Al-Tai et al. 1999) and *S. solisilvae* (Zhou et al. 2017).

The genomic features of the isolate, *S. albiflaviniger* DSM 41598^T^ and *S. javensis* DSM 41764^T^ are shown in Table 1. It is interesting that these strains have draft genomes over 8 Mbp in size and hence can be considered to be gifted after Baltz (2017). Available whole genome sequences of type strains of species classified in the *S. violaceusniger* 16S rRNA gene clade have larger genome sizes, as exemplified by *S. iranensis* HM 35^T^ (12.1 Mb; Horn et al. 2014) and *S. rapamycinicus* (12.7 Mb; Baranasic et al. 2013), the genome of the latter contains 48 BGCs including the biocluster expressing for rapamycin biosynthesis.

**Table 1:** Genomic features of the isolate and type strains of *S. albiflaviniger* and *S. javensis*

Genomic features	Isolate PRKS01-29^T^	*S. albiflaviniger* DSM 11483^T^	*S. javensis* DSM 41764^T^
Genome size (Mbp)	10.2	10.3	11.1
Mean coverage	56.92	9.93	35.33
Number of contigs	1104	3530	1486
Number of rRNA operons	8	8	8
Number of tRNA operons	64	59	71
G+C (mol%)	71.66	70.90	71.23
GenBank accessions	JAEEAP000000000.1	JAEEAR000000000.1	JAEEAQ000000000.1

**Fig. 1:**
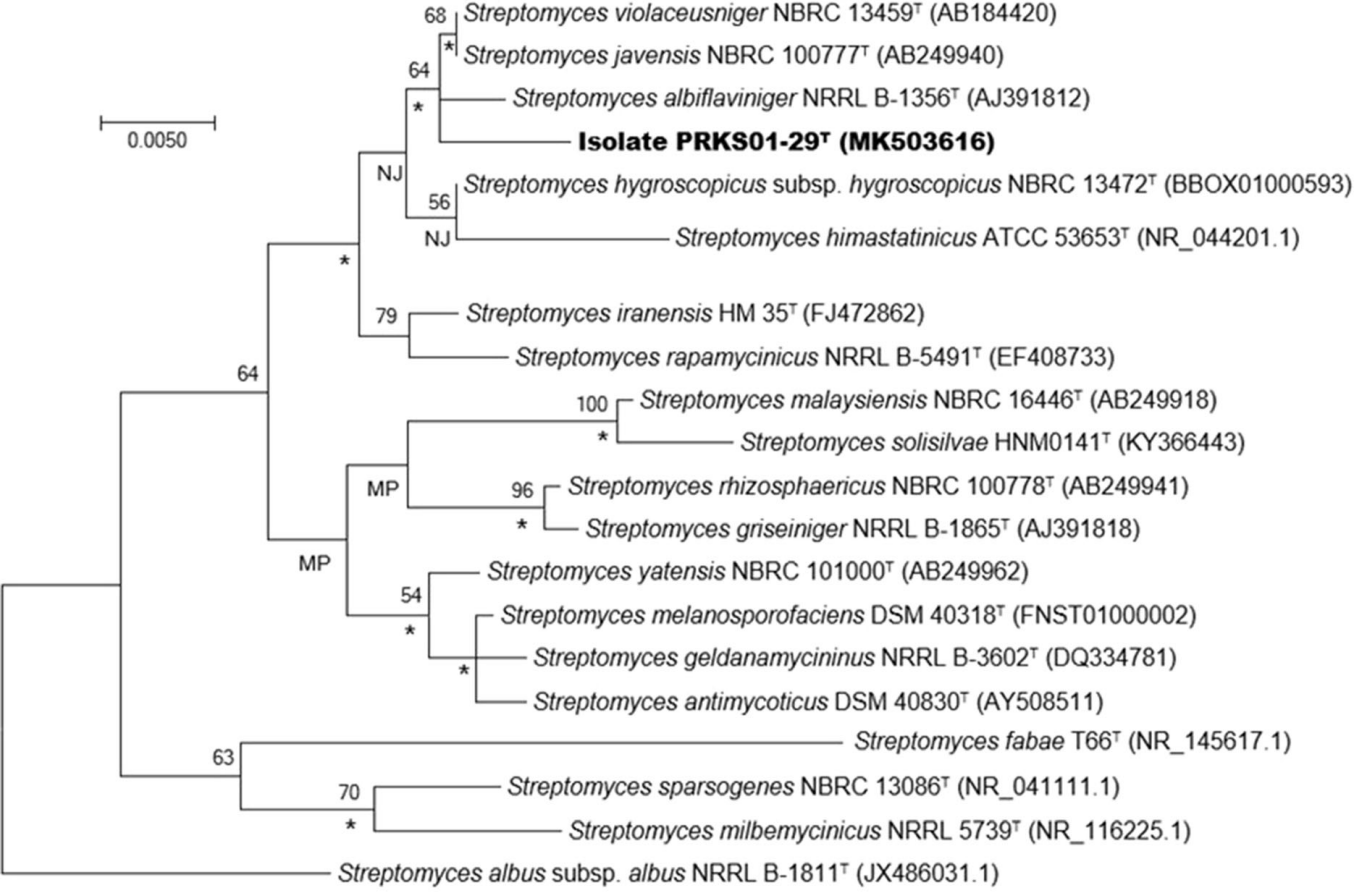
Maximum-likelihood tree based on 16S rRNA gene sequences showing relationships between isolate PRKS01-29^T^ and closely related type strains of *Streptomyce*s species classified in the *Streptomyces violaceusniger* clade. Asterisks indicate branches of the tree that were found using the neighbour-joining and maximum-parsimony algorithms. NJ and MP denote nodes recovered using the neighbour-joining and maximum-parsimony tree-making algorithms, respectively. Numbers at the nodes show bootstrap values, only those above 50% are shown. The root of the tree was established using *Streptomyces albus* subsp. *albus* NRRL B-1811^T^. Bar indicates 0.005 substitutions per nucleotide position

The phylogenetic tree (Fig. 1) based on 16S rRNA gene sequences shows that the isolate forms a clade in the *Streptomyces* gene tree together with the type strains of *S. albiflaviniger*,* S. javensis* and *S. violaceusniger*. It is most closely related to *S. javensis* NBRC 100777^T^ and *S. violaceusniger* NBRC 13459^T^ sharing a similarity with these strains of 99.4%, a value which corresponds to 9 nucleotide (nt) differences., the corresponding values with *S. albiflaviniger* NRRL B-1356^T^ are 99.3% (10 nt differences in 1414 sites). The 16S rRNA gene similarities between the isolate and the remaining representatives of the *S. violaceusniger* clade were within the range 96.8% to 99.2%. In general, these results are in agreement with those reported by Labeda et al. (2012) who found that streptomycetes producing spores with rugose or rough surfaces belonged to six highly related clades.

**Fig. 2:**
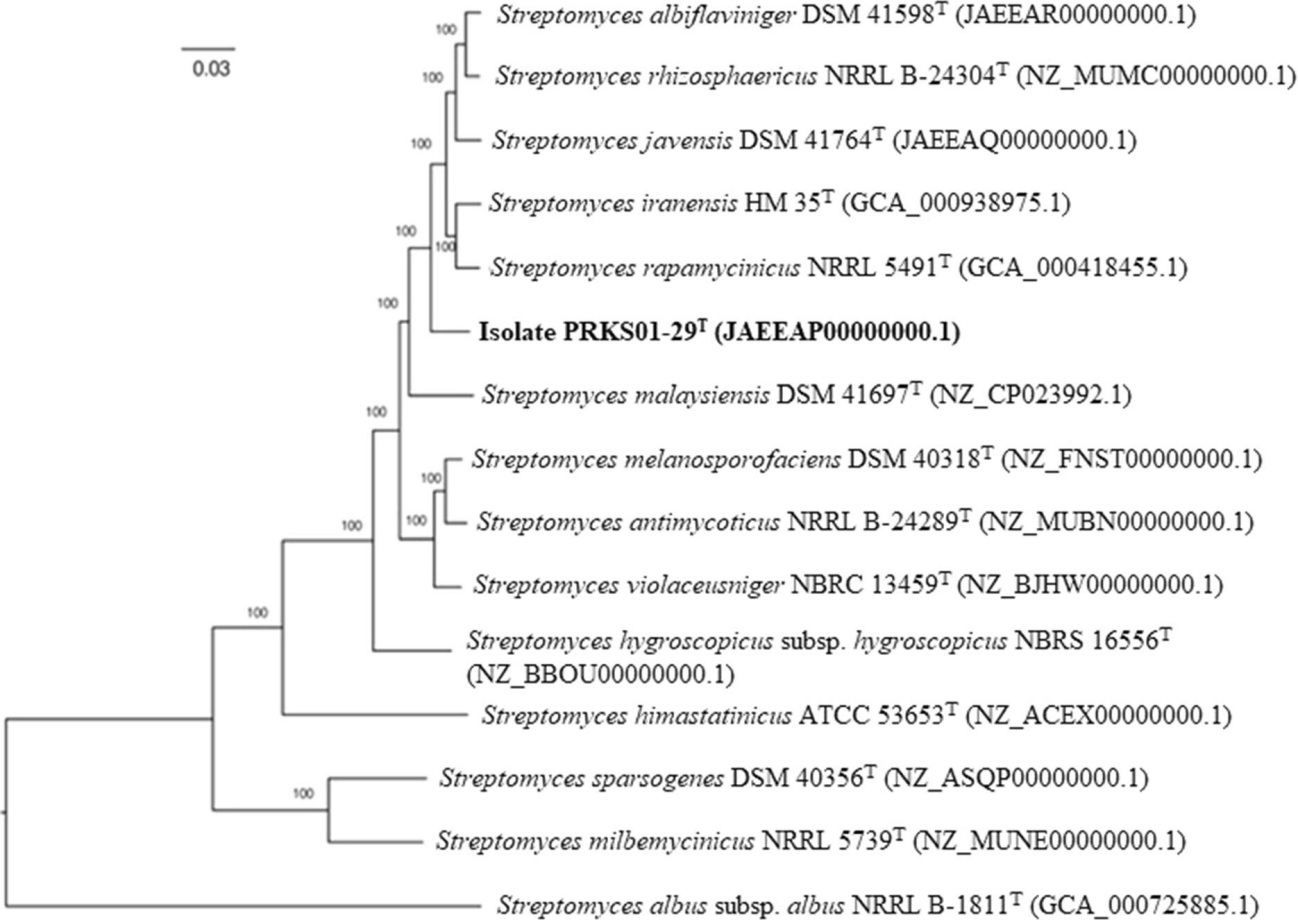
Maximum-likelihood phylogenomic tree based on 453 single copy core genes showing relationships between isolate PRKS01-29^T^ and closely related type strains which belong to the *Streptomyces violaceusniger* clade. Numbers at the nodes are bootstrap support values based on 100 replicates. GenBank accession numbers are shown in parentheses. The scale bar indicates 0.03 substitutions per nucleotide position. The tree is rooted using the type strain of *Streptomyces albus* subsp. *albus*

The phylogenomic tree (Fig. 2) shows that the isolate forms a distinct branch at the periphery of a subclade that encompasses the type strains of *S. albiflaviniger*,* S. iranensis*,* S. javensis*,* S. rapamycinicus* and *S. rhizosphaericus*. The *S. malaysiens*is strain form a distinct lineage between this and a sister subclade composed of the type strains of *S. antimycoticus*,* S. melanosporofaciens* and *S. violaceusniger*. The two remaining members of the *S. violaceusniger* clade, *S. himastatinicus* ATCC 58653^ T^ and *S. hygroscopicus* subspecies *hygroscopicus* NBRC 16556^T^ form single membered lineages. The close phylogenomic relationships between the type strains of *S. milbemycinicus* and *S. sporogenes* and *S. violaceusniger* clade is in agreement with the earlier study by Nouioui et al. (2018).

The recommended thresholds used to distinguish between closely related prokaryotic species based on ANI and dDDH similarities are 95 to 96% (Richter and Rosselló-Móra 2009; Chun et al. 2018) and 70% (Meier-Kolthoff 2013a; Chun et al. 2018), respectively. Table 2 shows that on this basis the isolate can be separated from the type strains of its closest phylogenomic neighbours, as shown in Fig. 2. It is most closely related to *S. albiflaviniger* DSM 41598^T^ based on a dDDH similarity of 53.9% and an ANI value of 93.5% though this latter value is shared with *S. javensis* DSM 41764^T^ and *S. iranensis* HM 35^T^.

**Table 2:** Average nucleotide identities and digital DNA:DNA hybridisation values between the isolate and *Streptomyces* species belonging to the *S. violaceusniger* clade

Phylogenomic neighbours	ANI (%)	dDDH (%)
*S. albiflaviniger* DSM 41598^T^	93.5	53.9
*S. antimycoticus* NRRL B-24289^T^	91.3	44.7
*S. himastatinicus* ATCC 53653^T^	84.9	29.0
*S. hygroscopicus* subsp. *hygroscopicus* NBRC 16556^T^	90.8	41.1
*S. iranensis* HM 35^T^	93.5	52.0
*S. javensis* DSM 41764^T^	93.5	52.8
*S. malaysiensis* DSM 41697^T^	91.6	44.4
*S. melanosporofaciens* DSM 40318^T^	91.5	44.9
*S. rapamycinicus* NRRL 5491^T^	93.4	51.1
*S. rhizosphaericus* NRRL B-24034^T^	93.3	52.6
*S. violaceusniger* NBRC 13459^T^	93.7	52.7

Identical results were obtained for the duplicated cultures in all of the phenotypic tests. It is also encouraging that the results of the biochemical, degradative and tolerance tests are in agreement with those from earlier analyses on the reference strains that were performed under the same experimental procedures (Al-Tai et al. 1999; Sembiring et al. 2000; Saintpierre et al. 2003; Goodfellow et al. 2007; Kumar and Goodfellow 2008; Hamedi et al. 2010; Zhou et al. 2017). Table 3 shows that the isolate can be separated from the type strains of all of its closest phylogenomic neighbours using a combination of phenotypic properties. It can, for instance, be distinguished from *S. albiflaviniger* DSM 14548^T^, its closest neighbour, as it is positive for esterase (C4), α-glucosidase and lipase (C14), casein, Tween 20 and uric acid, hydrolyses allantoin and grows in the presence of 7% w/v NaCl. In contrast, the *S. albiflaviniger* strain, unlike the isolate, hydrolyses arbutin. Additional combinations of phenotypic properties distinguish the isolate from the remaining reference strains and also the latter from one another.

**Table 3:** Phenotypic characteristics which distinguish isolate PRKS01-29^T^ from the type strains of closely related species classified in the *Streptomyces violaceusniger* clade

Characteristics	Strains						
	1	2	3	4	5	6	7
*API-ZYM tests*							
Esterase (C4)	+	−	−	+	+	+	+
α-Fucosidase	−	−	−	−	+	−	+
α- and β-Galactosidase, α-mannosidase, trypsin	+	+	+	+	+	−	+
β-Glucuronidase	−	−	−	+	−	−	−
α-Glucosidase	+	−	−	−	−	−	−
β-Glucosidase	−	−	+	+	−	−	−
Lipase (C14)	+	−	+	−	−	+	−
*Biochemical tests*							
Nitrate reduction	−	−	−	+	−	+	+
*Degradation tests*							
Adenine (0.5%, w/v)	+	+	−	+	+	+	+
Aesculin (0.1%, w/v)	−	−	+	+	+	−	+
Allantoin (0.5%,w/v)	+	−	+	+	−	+	−
Arbutin (0.5%, w/v)	−	+	+	+	−	+	−
Casein (1%, w/v)	+	−	+	+	+	−	+
Guanine (0.3%, w/v)	−	−	−	+	−	+	−
Starch (0.1%, w/v)	+	+	+	+	+	−	−
Tween 20 (1%, v/v)	+	−	+	+	+	−	−
Uric acid (0.4%, w/v)	+	−	+	+	+	+	−
Xylan (0.4%, w/v)	+	+	+	+	−	+	+
*Tolerance tests*							
Growth in presence of 7% w/v, NaCl	+	−	−	+	+	+	−
Growth at pH 9.0	−	−	+	–	+	+	−
Growth at 45 °C	+	+	−	+	−	−	+

Strains: 1. Isolate PRKS01-29^T^., 2. *S. albiflaviniger* DSM 14598^T^., 3. *S. iranensis* DSM 41954^T^., 4. *S. javensis* DSM 41764^T^., 5. *S. rapamycinicus* NRRL 5491^T^., 6. *S. rhizosphaericus* NRRL B-24304^T^., 7. *S. violaceusniger* DSM 40563^T^

All of the strains were positive for acid and alkaline phosphatases, α-chymotrypsin, cystine, leucine and valine arylamidases, esterase (C4), esterase lipase (C8), N-acetyl-β-glucoronidase and naphthol-AS-BI-phosphohydrolase (API-ZYM tests), hydrolysed urea and degraded hypoxanthine (0.4%, w/v), Tweens 40, 60 and 80 (all 1%, v/v) and L-tyrosine (0.4%, w/v), but not chitin (1%, w/v), elastin (0.3%, w/v), tributyrin (0.1%, w/v) or xanthine (0.4%, w/v)

^+^ positive., − negative, n.d. not determined

The aerial spore mass and substrate mycelial colours produced by the respective reference strains on the ISP media are in agreement with those from earlier analyses (Al-Tai et al. 1999; Goodfellow et al. 2007; Kumar and Goodfellow 2008; Hamedi et al. 2010). Table S2 shows that the isolate and its closest phylogenomic neighbours grew well on nearly all of the ISP media forming a grey-yellowish substrate mycelium bearing a grey aerial spore mass that became moist and black on prolonged incubation on ISP 3 agar, as is the case with the type strains of *S. antimycoticus* (Kumar and Goodfellow 2008; Komaki and Tamura 2020a), *S. griseiniger* (Goodfellow et al. 2007), *S. hygroscopicus* (Labeda and Lyons 1991) and *S. yatensis* (Saintpierre et al. 2003). The isolate and the *S. albiflaviniger* can be distinguished by their ability to produce diffusible pigments, for instance, only the reference strain produced diffusible pigments on ISP media 3 and 7.

The isolate showed activity in the primary and secondary screens. Growth of the *S. aureus* strain was inhibited when the isolate was grown on ISP 2, ISP 3, MMM and 410 agar media. Similarly, it inhibited the *B. subtilis, C. albicans* and *M. luteus* strains following cultivation on all of the nutrient formulations, apart from medium 410. In contrast, it did not show any activity against the *E. coli* strain though it did inhibit the growth of the *P. aeruginosa* strain when grown on ISP 3 and MMM agar. In the secondary screens, the isolate formed blue halos around inhibition zones against *B. subtilis* reporter strains YpuA^ER^, YvqI^ER^, Yjax^ER^ and DinB^CH^ indicating its ability to inhibit cell envelope, DNA, fatty acid and RNA synthesis, respectively. It also inhibited the growth of the other reporter strains, YvgS^ER^ and YheH, without forming blue halos thereby suggesting an ability to produce bioactive compound(s) with unknown modes of action.

### Biosynthetic potential of isolate PRKS01-29^T^ and members of the *S. violaceusniger* clade

The isolate and the type strains of species classified in the *S. violaceusniger* clade have large genomes (10.1–12.7 Mb) predicted to encode for chemically diverse specialised metabolites. The genome mining studies showed that all of the strains are genetically equipped with bioclusters predicted to encode for ‘core secondary’ metabolites, such as albaflavenone/geosmin, ectoines, hopenes, melanin and spore pigments, results in good agreement with those of Ward and Allenby (2018). In contrast, most of the bioclusters predicted to encode for druggable molecules, notably antibiotics, were discontinuously distributed in the genomes of the strains with many being strain specific, as has been found in recent studies on streptomycetes (Vicente et al. 2018; Martinet et al. 2020).

The genome of all of the strains contained bioclusters predicted to encode for echosides A-E, anti-tumor agents produced by *Streptomyces* strain LZ35 (Zhu et al. 2014). In contrast, only the genomes of the isolate and the type strains of *S. iranensis*,* S. violaceusniger* and *S. rapamycinicus* contained bioclusters considered to express for meilingmycin, an anti-parasitic macrolide (Sun et al. 2002) and nigericin, which inhibits Gram-positive bacteria (Graven et al.^1966^). Similarly, the draft genomes of the isolate, *S. albiflaviniger* DSM 41598^T^ and *S. javensis* DSM 41764^T^ contained bioclusters predicted to encode for the synthesis of cahuitamycins A-C, which inhibit the formation of bacterial biofilms (Park et al. 2016), pladienolides, anti-tumour antibiotics (Mizui et al. 2004) and funisamine, an aminopolyol polyketide antibiotic which inhibits the growth of wild type strains of *Staphylococcus aureus*,* Escherichia coli* and *Candida albicans* (Covington et al. 2018), respectively. Bioclusters predicted to encode for rapamycin were only detected in the genomes of the *S. iranensis* and *S. rapamycinicus* strains.

## Conclusion

It can be concluded from the phylogenetic trees and associated colonial and morphological data that isolate PRKS01-29^T^ belongs to the *S. violaceusniger* clade (Sembiring et al. 2000; Goodfellow et al. 2007; Kumar and Goodfellow 2008, 2010). In addition, the whole genome sequence data show that it belongs to a well-supported monophyletic clade which includes the type strains of *S. albiflaviniger*,* S. iranensis*,* S. javensis*,* S. rapamycinicus* and *S. rhizosphaericus*. It can be distinguished from all of these strains by a broad range of phenotypic properties and by low ANI and dDDH values. It is, therefore, proposed that isolate PRKS01-29^T^ represents a novel species within the genus *Streptomyces* for which the name *Streptomyces sabulosicollis* sp. nov. is proposed.

### Description of *Streptomyces sabulosicollis* sp. nov.

*Streptomyces sabulosicollis* (sa.bu.lo.si.col’lis. L. masc. adj. *sabulosus* sandy; L. masc. n. *collis* a hill; N.L. gen. n. *sabulosicollis* of a sandy hill), Gram-stain-positive, catalase positive, aerobic actinobacterium which forms an extensively branched substrate mycelium and aerial hyphae which differentiate into spiral chains of rugose ornamented spores (0.8 × 0.97 µm) on yeast extract-malt extract agar. A yellowish-grey substrate mycelium carries a grey aerial spore mass that becomes moist and black following prolonged incubation on oatmeal agar. Grows from 10 to 45 °C (optimally at 28 °C), from pH 5.5–7.5 (optimally 7.0) and can tolerate up to 7% (w/v) NaCl. Allantoin and urea are hydrolysed but not aesculin or arbutin. Does not reduces nitrate. Degrades adenine, casein, hypoxanthine, starch, L-tyrosine, Tweens 20, 40, 60 and 80, uric acid and xylan, but not chitin, elastin, guanine, tributyrin or xanthine. Positive for acid and alkaline phosphatases, α-chymotrypsin, cystine, leucine and valine arylamidases, esterase lipase, α- and β-galactosidases, α-glucosidase, N-acetyl-β-glucosidase, lipase (C14), α-mannosidase, naphthol-AS.BI-phosphohydrolase and trypsin, but not α-fucosidase, β-glucosidase or β-glucuronidase. Whole organism hydrolysates contain LL-A_2_pm, galactose, glucose, mannose and ribose., the predominant fatty acids (> 10%) are *iso*-C_15:0_ (14.4%), *anteiso*-C_15:0_ (13.8%) and *iso*-C_16:0_ (27.2%), the major menaquinones MK-9 (H6, H8) with the proportions of 58.4% and 41.6%, respectively, and the polar lipid profile is composed of diphosphatidylglycerol, two phosphatidylglycerols, phosphatidylinositol, two phosphatidylinositol mannosides and two unknown phospholipids. The dDNA G+C content of the strain is 71.7% and its approximate genome size 10.2 Mbp.

The type strain, PRKS01-29^T^ (= CCMM B1303^T^ = ICEBB-02^T^ = NCIMB 15210^T^) was isolated from a sandy soil sample collected from an arid sand dune system in the Parangkusumo Region of Yogyakarta Province, Java, Indonesia. The GenBank accession number of the assembled draft genome of *Streptomyces sabulosicollis* is JAEEAP000000000.1.

In the case of the genus *Streptomyces* genome-based classifications have revealed the presence of well-defined species-groups (Labeda et al. 2012, 2017; Nouioui et al. 2018), the recognition of later heterotypic synonyms of established species (Komaki and Tamura 2020a, b; Madhaiyan et al. 2020) within and outwith the *S. violaceusniger* phylogenetic clade (Sembiring et al. 2000; Goodfellow et al. 2007; Kumar and Goodfellow 2008, 2010) and the delineation of the genera *Embleya* and *Yinghuangia* for species previously included in the genus (Nouioui et al. 2018). Such developments can be expected to continue and in this respect, it is evident from this study that streptomycetes which form rugose-ornamented spores, spiral spore chains and characteristic colonial properties on oatmeal agar belong to a distinct phylogenomic clade the taxonomic status of which merits further investigation.

## Data availability statements

The 16S rRNA gene and whole genome sequences of strain PRKS01-29^T^ that support the findings of this study have been deposited in GenBank database with the accession numbers MK503616 and JAEEAP000000000.1, respectively. In turn, corresponding accession numbers for the whole genome sequences of *Streptomyces albiflaviniger* DSM 42598^T^ and *Streptomyces javensis* DSM 41764^T^ are JAEEAR000000000.1 and JAEEAQ000000000.1, respectively. All the whole genome sequences described in this paper is version 1.

## Supplementary Information

Below is the link to the electronic supplementary material.

Supplementary file1 (PDF 315 kb)
