# Supplementary material for: Genome-based classification of the Streptomyces violaceusniger clade and description of Streptomyces sabulosicollis sp. nov. from an Indonesian sand dune
Source: Antonie Van Leeuwenhoek. 2021 Apr 2;114(6):859–73. doi: 10.1007/s10482-021-01564-0 (PMC8137480; doi:10.1007/s10482-021-01564-0)
Supplement: Supplementary file 1 — Supplementary file1 (PDF 315 kb) [file 10482_2021_1564_MOESM1_ESM.pdf]

Supplementary Data

Genome-based classification of the *Streptomyces violaceusniger* clade and description of *Streptomyces sabulosicollis* sp. nov. from an Indonesian sand dune

Ali B. Kusuma, Imen Nouioui and Michael Goodfellow

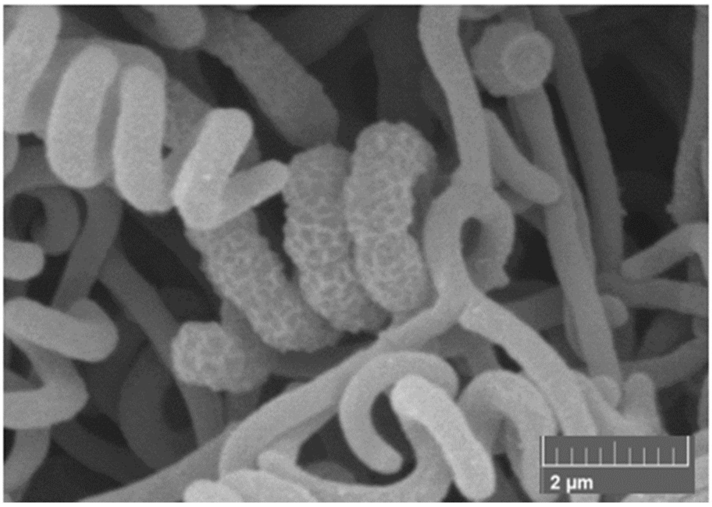

Figure S1. Scanning electron micrograph of isolate PRKS01-29^T showing spiral spiral chains of rugose ornamented spores following growth on ISP 3 at 28°C for 14 days. Bars: 2 µm.

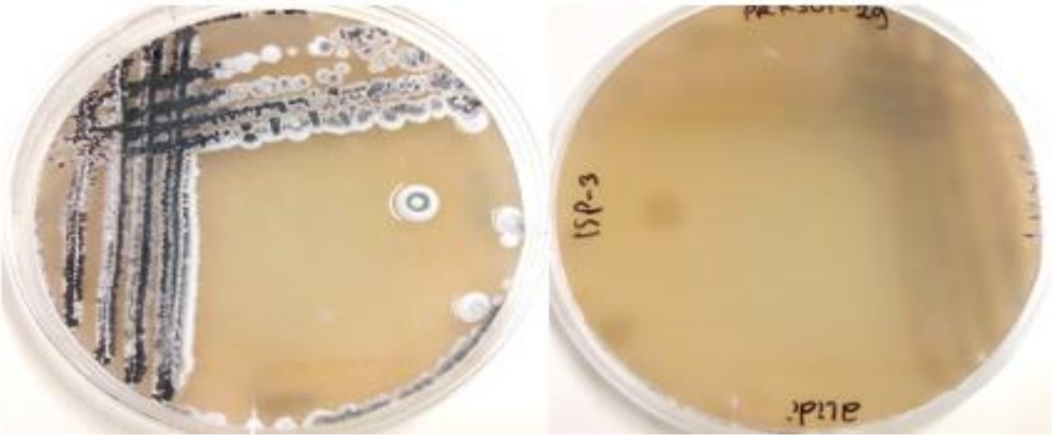

Figure S2. Cultural characteristic of isolate PRKS01-29^T showing a grey aerial spore mass which turned black and moist (left) and a greyish yellow substrate mycelium (right) following growth on ISP 3 after 21 days at 28°C.

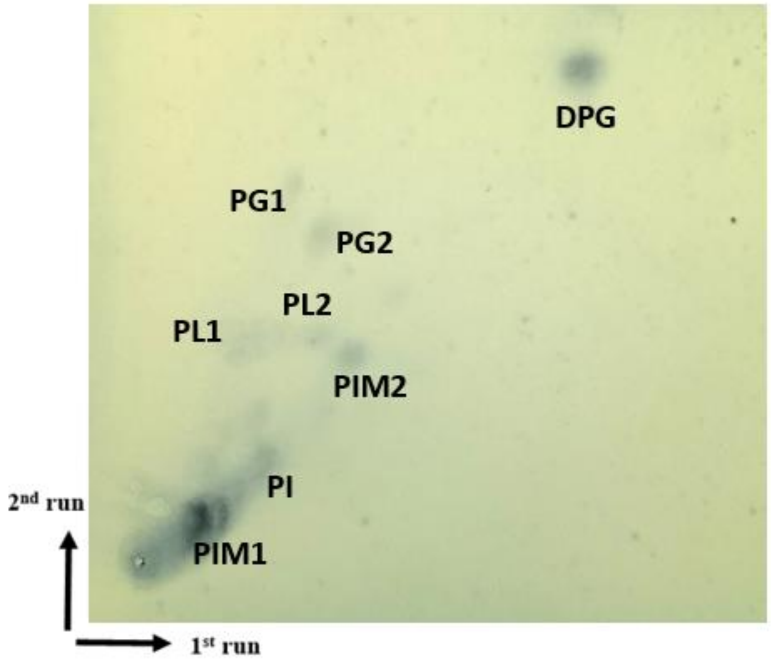

Figure S3. Two-dimensional thin-layer chromatography of the polar lipids of isolate PRKS01-29^T stained with molybdatophosphoric acid spray (Sigma P1518). Key: DPG, diphosphatidylglycerol; PG1 and 2, phosphatidylglycerols; PI, phosphatidylinositol; PIM1 and 2, phosphatidylinositolmannosides; PL1 and 2, unidentified phospholipids. Solvent 1: chloroform: methanol: distilled water (65:25:4 v/v); Solvent 2: chloroform: glacial acetic acid: methanol: distilled water (80:12:15:4 v/v). In the first run the TLC plate was positioned in a perpendicular position towards the solvent direction., in the second run, the position of the TLC plate and solvent were in the same direction.

Table S1. *Bacillus subtilis* reporter strains and positive controls used in plug assays designed to determine modes of action of antimicrobial compound(s) produced by isolate PRKS01-29^{T}

Reporter Strains	Targets	Positive Controls
YvqI^{ER}	Cell wall synthesis	Bacitracin
YpuA^{ER}	Cell envelope synthesis	Cefoxitin
DinB^{CH}	DNA synthesis	Nalidixic acid
Yjax^{ER}	Fatty acid synthesis	Triclosan
YvgS^{ER}	RNA synthesis	Rifampicin
YheH	Sporulation	Tetracycline

Er, erythromycin resistant; CH, chloramphenicol resistant.

Table S2. Growth and cultural features of isolate PRKS01-29^{T} and reference strains following incubation at 28°C for 14 days.

Strains	Growth and cultural characteristics	ISP media						
		1	2	3	4	5	6	7
Isolate PRKS01-29^{T}	Growth	+++	+++	+++	+++	+/-	+	+++
	Aerial spore mass	White	Greyish-black	Greyish-black	White	White grey	Light yellow	Greyish-black
	Substrate mycelium	White	Dark brown	Greyish-yellow	Light brown	Colourless	Light yellow	Dark brown
	Diffusible pigments	None	None	None	Brown	None	None	None
S. albiflaviniger DSM 41598^{T}	Growth	+++	+++	+++	+++	+	+	+++
	Aerial spore mass	White	White-grey	White	White	White grey	White	White
	Substrate mycelium	White	Dark brown	Yellow	Orange-grey	Colourless	White	Medium brown
	Diffusible pigments	None	None	Orange	Orange	None	None	Yellow
S. iranensis DSM 41954^{T}	Growth	+++	+++	+++	+++	+++	+++	+++
	Aerial spore mass	Beige	Beige	Grey	Grey	White	Brown beige	Brown beige

	Substrate mycelium	Yellow-brownish	Yellow-brownish	Yellow-brownish	Yellow-brownish	Yellow-brownish	Yellow-brownish	Yellow-brownish
S. javensis DSM 41764^T	Growth	+++	+++	+++	+++	++	+++	+++
	Aerial spore mass	Beige	Medium grey	Grey	White	White	Beige	Black
	Substrate mycelium	White	Dark brown	Greyish-yellow	Light brown	White	Beige	Dark brown
	Diffusible pigments	None	None	Yellow	None	None	None	None
S. rapamycinicus NRRL 5491^T	Growth	+++	+++	+++	+++	+++	+++	+++
	Aerial spore mass	Beige	Greyish black	Grey	Grey	Cream	Beige	Cream
	Substrate mycelium	Beige	Beige	Greyish-yellow	Beige	Beige	Beige	Beige
S. rhizosphaericus NRRL B-24304^T	Growth	+++	+++	+++	+++	++	+++	+++
	Aerial spore mass	Beige	Greyish black	Grey	Grey	Cream	Beige	Cream
	Substrate mycelium	Beige	Beige	Greyish-yellow	Beige	Beige	Beige	Beige
	Diffusible pigments	None	None	Yellow	None	None	None	None
	Growth	+++	+++	+++	+++	+++	++	+++

S. violaceusniger DSM 40563^T	Aerial spore mass colour	White	Dark grey	Grey	White	White-grey	White	Medium grey
	Substrate mycelium colour	White	Dark brown	Greyish-yellow	Light brown	Greyish-yellow	White	Medium brown

Growth: +++, abundant., ++ moderate., + poor growth., - no growth. Diffusible pigments were not formed by the *S. iranensis, S. rapamycinicus* and *S. violaceusniger* strains.

ISP media : 1, tryptone-yeast extract., 2, yeast extract-malt extract., 3, oatmeal., 4, inorganic-salts-starch., 5, glycerol-asparagine., 6, peptone-yeast extract-iron and 7, tyrosine agar (Shirling and Gottlieb 1966).
